# The impact of technological innovation on the green digital economy and development strategies

**DOI:** 10.1371/journal.pone.0301051

**Published:** 2024-04-25

**Authors:** Yanlin Liu, Yaoguang Yang, Xiyue Zhang, Yaohui Yang

**Affiliations:** 1 School of Law, Zhejiang University, Hang Zhou City, China; 2 College of Human Health Sciences, University College London, London, Britain; 3 Department of Media Communication and Cultural Studies, Goldsmiths University of London, London, Britain; 4 School of HNU·ASU Joint International Tourism College, Hainan University, Haikou, China; University of Central Punjab, PAKISTAN

## Abstract

To investigate the interplay among technological innovation, industrial structure, production methodologies, economic growth, and environmental consequences within the paradigm of a green economy and to put forth strategies for sustainable development, this study scrutinizes the limitations inherent in conventional deep learning networks. Firstly, this study analyzes the limitations and optimization strategies of multi-layer perceptron (MLP) networks under the background of the green economy. Secondly, the MLP network model is optimized, and the dynamic analysis of the impact of technological innovation on the digital economy is discussed. Finally, the effectiveness of the optimization model is verified by experiments. Moreover, a sustainable development strategy based on dynamic analysis is also proposed. The experimental results reveal that, in comparison to traditional Linear Regression (LR), Decision Tree (DT), Random Forest (RF), Support Vector Machine (SVM), and Naive Bayes (NB) models, the optimized model in this study demonstrates improved performance across various metrics. With a sample size of 500, the optimized model achieves a prediction accuracy of 97.2% for forecasting future trends, representing an average increase of 14.6%. Precision reaches 95.4%, reflecting an average enhancement of 19.2%, while sensitivity attains 84.1%, with an average improvement of 11.8%. The mean absolute error is only 1.16, exhibiting a 1.4 reduction compared to traditional models and confirming the effectiveness of the optimized model in prediction. In the examination of changes in industrial structure using 2020 data to forecast the output value of traditional and green industries in 2030, it is observed that the output value of traditional industries is anticipated to decrease, with an average decline of 11.4 billion yuan. Conversely, propelled by the development of the digital economy, the output value of green industries is expected to increase, with an average growth of 23.4 billion yuan. This shift in industrial structure aligns with the principles and trends of the green economy, further promoting sustainable development. In the study of innovative production methods, the green industry has achieved an increase in output and significantly enhanced production efficiency, showing an average growth of 2.135 million tons compared to the average in 2020. Consequently, this study highlights the dynamic impact of technological innovation on the digital economy and its crucial role within the context of a green economy. It holds certain reference significance for research on the dynamic effects of the digital economy under technological innovation.

## Introduction

In today’s world, with accelerating globalization and rapid technological development, the digital economy has become a key force driving economic growth. However, with the swift economic growth and rapid industrial development, environmental problems have become increasingly prominent, such as climate change, resource depletion, and ecological destruction, etc., pose a major challenge to the sustainable development of society (Li and Wang, 2022, [1]). In this context, the concept of a green economy came into being, and its core goal is to achieve a win-win situation between economic growth and environmental sustainability. The green economy emphasizes economic transformation and upgrading by promoting the use of clean energy, improving resource efficiency, and promoting the innovation and application of environmentally friendly technologies. Therefore, it has become an urgent and important topic to study the dynamic impact of scientific and technological innovation on the digital economy under the background of green economy and explore its sustainable development strategy. This study’s research background and motivation come from a deep understanding of the current global environmental crisis and the need for economic transformation (Zhang et al., 2021; Ding et al., 2021; Luo et al., 2023, [2–4]). On the one hand, environmental degradation poses a threat to human survival and social development, and it is urgent to solve this problem through scientific and technological innovation and industrial restructuring. On the other hand, with the fast growth of information technology, the digital economy has shown great growth potential and broad development prospects and has become a new engine for promoting economic and social development. However, the rapid expansion of the digital economy has also brought new challenges, including increased energy consumption, e-waste generation, etc., which need to be properly addressed within the framework of the green economy.

Therefore, this study aims to explore in depth how to promote the sustainable development of the digital economy through scientific and technological innovation in the context of the green economy, while considering the balance of economic growth, environmental protection, and resource efficiency. Its motivation is to identify and address the environmental and social challenges in the development of the digital economy, promote the green transformation and upgrading of the industrial structure by optimizing technology and production methods, and achieve economic growth while reducing the negative impact on the environment. In this way, this study not only seeks to promote the harmonious coexistence of the economy and the environment but also provides policymakers, industry, and academia with strategies and solutions on how to achieve sustainable development of the digital economy. The study begins by analyzing the limitations and optimization strategies for multi-layer perceptron (MLP) networks in the context of the green economy. It optimizes the MLP network model, discusses the dynamic analysis of the impact of technological innovation on the digital economy, and validates the effectiveness of the optimized model through experiments. Sustainable development strategies based on dynamic analysis are also proposed.

## Literature review

The green economy represents a model that simultaneously promotes economic growth, social progress, environmental protection, and sustainable development. In this model, technological innovation plays a central role, prompting exploration into its profound implications for the dynamics of the digital economy. Existing research has validated the intricate interrelationship between the green economy and the digital economy, emphasizing the indispensable contribution of technological innovation to driving the development of the digital economy. Zhang et al. (2022) delved into the interaction between the green economy and the digital economy, revealing that the green economy propelled the development of the digital economy, while the digital economy, in turn, supported the green economy by providing technological support and innovative pathways. This finding unveiled the interactive relationship between the two and underscored their mutual interdependence in promoting sustainable development (Zhang et al., 2022a, [5]). Zhao et al. (2023) conducted a study on the dynamic impact of technological innovation on the digital economy, elucidating how advancements in artificial intelligence (AI), blockchain, and the Internet of Things (IoT) drive digital economic growth and profoundly influence its industrial structure, production methods, and market allocation. This research expanded the understanding of the impact of technological innovation and emphasized innovation’s critical role in shaping the digital economy’s future (Zhao et al., 2023, [6]). Ma and Zhu (2022) focused on technological innovation in the elements of the green economy, such as clean energy and renewable resources, clarifying the core role of technological innovation in enhancing the efficiency and sustainable utilization of these components of the green economy. This analysis not only highlighted the importance of technological innovation in promoting resource-efficient utilization but also pointed out the critical role of innovation in achieving the coordinated development of economic and environmental goals (Ma and Zhu, 2022, [7]). Su et al. (2021) conducted a comprehensive review of relevant policies and legal frameworks. They established a carefully designed policy and legal structure that made significant contributions to promoting the coordinated development of technological innovation and the digital economy, thereby supporting the sustainable development of green and digital economies. This study highlighted the importance of the policy and regulatory environment in incentivizing and guiding technological innovation and economic transformation (Su et al., 2021, [8]). Yin and Zhao (2023) took rural new energy development as a starting point, conducting an in-depth analysis of the driving mechanisms behind rural new energy connections. They explored the relationships between different stakeholders, providing guidance for optimizing the construction of rural new energy systems. This research emphasized the significance of understanding and coordinating various stakeholders’ interests in advancing rural new energy development (Yin and Zhao, 2023, [9]). Yin et al. (2022) combined symbiosis theory with six analytical methods to innovatively construct a new framework for selecting joint investment partners in digital green innovation projects. By introducing a dual-combination weighted method, the study effectively avoided the subjective and objective biases of attribute weights and time weights. The empirical research validated the framework and selection model’s scientific, reliable, and practical nature. This achievement provided a new methodological framework for selecting suitable investment partners and offered practical guidance for the implementation of digital green innovation projects (Yin et al., 2022a, [10]). Yin et al. (2022) proposed a standard framework for ecological niche suitability assessment based on the niche theory and established an ecological niche domain model for innovation partner selection management based on this theory. The standard framework and novel niche domain model of this study provided vital theoretical support and practical guidance for businesses engaged in digital green innovation in high-end agricultural equipment development (Yin et al., 2022b, [11]).

Examining the dynamic repercussions of technological innovation on the digital economy within the framework of the green economy constitutes a crucial area of research. The impetus provided by technological innovation can steer the evolution and metamorphosis of the digital economy, necessitating the implementation of congruent sustainable development strategies to collectively advance the sustainability of the green digital economy.

Although studies on the impact of technological innovation on the digital economy in the context of the green economy have been initiated through previous research, most research remains at the level of static impact analysis, with insufficient exploration into the dynamic nature of this field. This has resulted in a lack of systematic research on sustainable development strategies for the integrated development of green and digital economies. Additionally, there is a need for strengthened assessment and promotion of strategies in practical applications. The current global economy is undergoing significant transformations due to environmental issues and technological advancements, making in-depth exploration in this field necessary and timely. This study makes remarkable innovations and expansions in several critical aspects compared to previous research. Firstly, it delves into the impact mechanism of technological innovation on the digital economy under the backdrop of the green economy, breaking through the limitations of previous studies and providing a new perspective for understanding the dynamic changes in this field. Furthermore, the study enriches related research content and proposes sustainable development strategies based on green technological innovation. These theoretically innovative strategies provide concrete paths and directions for the synergistic development of digital and green economies. In the current context of rapid global economic changes and escalating environmental challenges, the timeliness and innovativeness of this study hold significant theoretical and practical significance.

## Dynamic impact analysis model of MLP networks in the context of a green economy

### Limitations and optimization strategies of MLP networks in the context of green economy

The digital green economy is an emerging concept that combines the advantages of the digital economy and the green economy, aiming to promote environmental sustainability and economic growth through efficient digital technologies. The concept is an extension of the existing definition of the green economy, with particular emphasis on the vital role of digital technologies in promoting environmental protection and resource efficiency. The digital green economy encompasses the application of digital technologies to optimize resource use efficiency, reduce the environmental footprint, support the production and consumption of renewable energy, and promote innovation and development of green products and services. These areas range from smart energy management systems to green supply chains, from environmental monitoring and management technologies to sustainable agricultural technologies, as well as digital solutions that drive a circular economy and green finance.

As an advanced economic model integrating the green economy and digital technology concept, the digital green economy focuses on achieving the dual goals of environmental protection and economic growth through technological innovation. Key areas of this economic model include smart energy management, green supply chain and logistics, environmental monitoring and management, sustainable agriculture, circular economy, and green finance. At the same time, the importance of focusing on the digital green economy cannot be ignored. Firstly, it plays a key role in the fight against climate change, contributing to environmental protection by optimizing resource use and reducing greenhouse gas (GHG) emissions. Secondly, the digital green economy can promote economic growth by innovating green products and services. It is to create new economic growth points and optimize and upgrade the economic structure. In addition, it contributes to social well-being and promotes equitable and inclusive growth by improving the quality of the environment and enhancing the quality of life. The digital green economy also encourages technological innovation, developing new digital technologies and solutions to address environmental challenges and driving innovation and application of sustainable technologies. Finally, this area of research has vital implications for achieving the United Nations Sustainable Development Goals (SDGs), especially in clean energy, industrial innovation and infrastructure, sustainable cities and communities, and responsible consumption and production.

The MLP network represents a classical artificial neural network structure comprising multiple layers of neurons. It consists of an input, hidden, and output layer, wherein each layer’s neurons connect with all neurons in the preceding layer (Xue et al., 2022, [12]). Each neuron possesses specific weights and biases within the MLP network, determining its output through an activation function. Neurons in the input layer receive external input data, transmitting it to the hidden layers. Subsequently, the hidden layers undertake nonlinear transformations and feature extraction on the input data (Zhao et al., 2022, [13]). Ultimately, the output layer generates the conclusive results of the network. The training process of MLP networks commonly employs the backpropagation algorithm for weight updates (Zhang et al., 2022b, [14]). This algorithm computes the error between predictions and actual labels, optimizing through gradient descent based on this error to iteratively adjust the network’s weights and biases. This iterative adjustment facilitates the network in making more precise predictions (Sorescu and Schreier, 2021; Li et al., 2021b, [15, 16]). Traditional optimization methods encounter specific limitations when applied to MLP networks, as exhibited in Table 1:

**Table 1:** Limitations of traditional optimization methods in handling MLP networks.

Limit	Details
Vanishing gradient and exploding gradient problems	In deep MLP networks, the backpropagation algorithm reversely propagates errors layer by layer to update weights. However, as the number of network layers increases, the gradient may decay or grow exponentially, leading to gradient disappearance or explosion problems. This will cause the network to be unable to effectively learn effective feature representations, limiting the network’s expressive ability and training effect.
Computing resource requirements are high.	Traditional optimization methods require much computing resources and time when training MLP networks on large-scale data sets. Since each sample needs to be calculated one by one for forward propagation and backpropagation, this will lead to longer training time and greater resource consumption.
local optimal solution problem	MLP networks are non-convex optimization problems with multiple local optimal solutions. Traditional optimization algorithms, such as gradient descent, are prone to falling into local optimal solutions and may be affected by initialization conditions, resulting in unstable network training results.

In the context of the green economy, this study considers the following strategies to optimize the energy consumption and efficiency of MLP networks, as outlined in Table 2:

**Table 2:** Optimization strategies for traditional MLP networks.

Optimization Strategy	Strategic Analysis
Energy saving optimization algorithm	Designing and applying energy-efficient optimization algorithms is key to optimizing multi-layer sensing networks in the green economy. The traditional gradient descent algorithm can improve energy efficiency through enhanced techniques by accelerating the training process, increasing the convergence speed, and reducing the consumption of computing resources.
Hardware acceleration technology	It uses specialized hardware accelerators to accelerate the calculation and training process of MLP networks. These hardware accelerators have high parallel computing capabilities and low power consumption characteristics, which can significantly improve computing and energy consumption efficiency.
Data processing and feature selection	It uses appropriate data preprocessing methods and feature selection techniques to reduce computing and storage requirements for large-scale data sets. Data dimensionality reduction, denoising, and feature selection can effectively reduce input data’s dimensionality and noise, improving training efficiency and model generalization ability.

**Fig 1:**
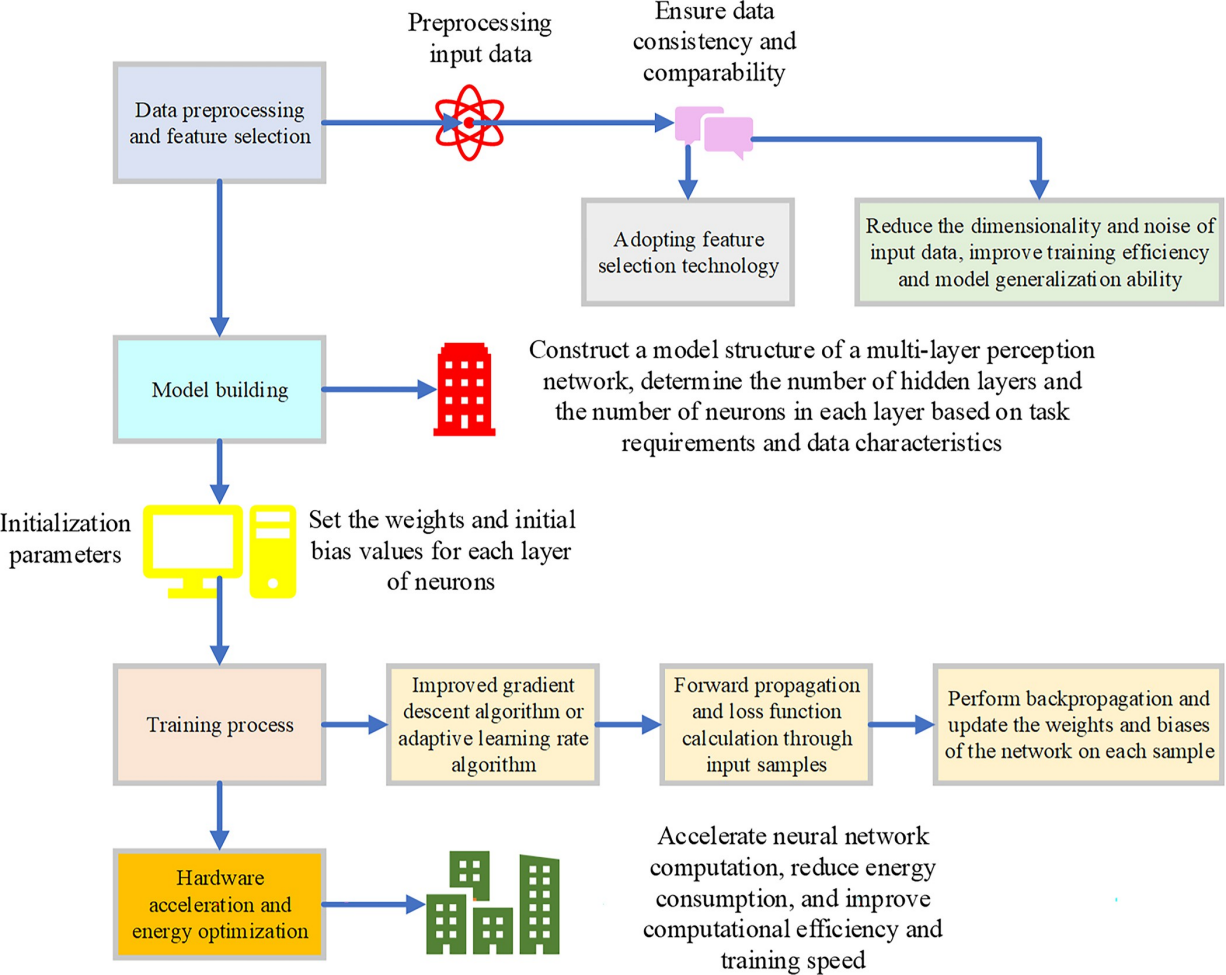
Optimized MLP network model.

By employing the aforementioned optimization strategies, proficient optimization of MLP networks can be attained within the framework of the green economy (Zhen et al., 2021; Li et al., 2021a; Shahbaz et al., 2022; Jun et al., 2021, [17–20]). This approach contributes to reducing energy consumption and mitigating environmental burdens and elevates the network’s performance and intelligence, thereby fostering the sustainable development of the digital economy. The post-optimized model architecture is illustrated in Fig 1:

In Fig 1, this study introduces energy-efficient optimization algorithms to reduce the model’s energy consumption and resource utilization. By dynamically adjusting parameters such as learning rates, pruning, and quantization, these algorithms aim to lower the computational and storage costs of the network. The application of these optimization algorithms can decrease redundant information transmission and computational loads between the perceptual layer and hidden layers, thereby enhancing the efficiency and energy performance of the model. To expedite the training and inference processes of the model, this study employs hardware acceleration techniques, including graphics processing unit (GPU) acceleration and dedicated hardware accelerators. Leveraging the parallel computing capabilities of these hardware accelerators significantly boosts the computational speed and efficiency of the MLP network, thereby expediting the model’s training and inference processes. Data processing and feature selection techniques are also utilized to optimize the MLP network model. Data processing techniques involve data preprocessing, standardization, normalization, etc., to enhance data usability and quality. Feature selection techniques involve filtering and selecting features from input data to reduce the impact of redundant information and extract the most discriminative features. This can reduce model complexity, improve generalization capability, and enhance classification performance. Compared to previous research, the model optimization in this study innovates in several aspects. Firstly, it introduces considerations for energy efficiency, reducing the model’s energy consumption and resource utilization through optimization algorithms and hardware acceleration techniques. Secondly, data processing and feature selection techniques improve the accuracy and generalization capability of the model. These optimization measures not only enhance the model’s performance but also make it more energy-efficient and environmentally friendly in practical applications, aligning with the development trend of the green economy.

### Dynamic impact analysis of technological innovation on the digital economy

With the intensification of global climate change and the increasing prominence of environmental issues, the green economy has become a critical path for countries to pursue sustainable development. Meanwhile, the rapid growth of the digital economy has profoundly transformed traditional economic models and social structures. In the context of the green and digital economies, technological innovation is vital in dynamically influencing the digital economy. It propels the development of the digital economy and provides new opportunities for realizing the green economy. Currently, the green and digital economies represent crucial trends in global development. The green economy, centered on sustainability, strives to balance economic growth and environmental protection. In contrast, the digital economy, based on technological innovation and digital transformation, propels digitization, intelligence, and networking in the economy. Studying the interrelationships and mutual influences of these two economic systems contributes to a deeper understanding of the evolution of the global economy and future development directions.

The digital economy is distinguished by its dependence on digital technology and information communication technology, with data, information, and knowledge constituting its fundamental resources. Within the digital economy, there has been a noteworthy augmentation in the transmission, storage, and utilization of information and data. The swift evolution and sustained innovation in digital technology propel economic transformation and growth (Wilson et al., 2022; Hao et al., 2023, [21, 22]). Data collection, processing, and application lie at the digital economy’s core. A substantial volume of data is consistently amassed and scrutinized to comprehend consumer needs and behavior, optimize product design, and refine business models (Grigorescu et al., 2021; Dabbous and Tarhini, 2021; Donnelly and Johns; 2021, [23–25]). The significance of data has evolved into a central asset in the digital economy (Ren et al., 2022; Sturgeon, 2021; Garud et al., 2022, [26–28]). The digital economy consistently nurtures innovation in both technological and business models. The amalgamation of digital technology and the internet propels the introduction of novel products, services, and business formats, furnishing fresh impetus for economic expansion (Chen et al., 2021, [29]). The digital economy disassembles barriers in traditional industries and encourages convergence and collaboration across diverse sectors (Meirun et al., 2021, [30]). It advocates principles of openness and sharing. Through the internet and digital technology, information and knowledge can be swiftly disseminated and shared, surmounting geographical constraints and facilitating collaboration on a global scale (Khan et al., 2021; Pouri and Hilty, 2021; Ciarli et al., 2021, [31–33]). Technological innovation stands out as a pivotal factor propelling the development of the digital economy. The role and impact of technological innovation on the digital economy are elucidated in Table 3.

**Table 3:** The role and impact of technological innovation on the digital economy.

Effect	Function analysis
Improve economic efficiency	Technological innovation can maximize economic benefits by increasing productivity, improving management methods, and optimizing resource allocation.
Promote business model innovation.	Technological innovation provides enterprises with new business models and opportunities. New business models emerge through the combination of digital technology and the Internet.
Expand market space	Technological innovation breaks the boundaries of traditional industries and opens up new market space.
Changes in industrial structure	The development and application of green technologies, such as clean energy, environmentally friendly processes, and sustainable development solutions, have promoted the transformation of traditional industries into low-carbon, environmentally friendly, and sustainable development directions. At the same time, emerging digital industries have also developed rapidly.
Innovation in production methods	Through technological means such as intelligence, automation, and digitalization, energy consumption and emissions during production have been reduced, and environmental impacts have also been mitigated.
The balance between economic growth and environmental impact	Scientific and technological innovation provides new solutions to environmental problems, such as new energy technologies, clean production technologies, etc., achieving a balance between economic growth and environmental protection.

Concurrently, technological innovation assumes a pivotal role in the digital economy, and a nuanced analysis of its dynamic impact on the digital economy is principally evident across several dimensions. Firstly, it augments productivity. Applying dynamic analytical techniques enables businesses to gain a more profound understanding of market demands, optimize resource allocation, and enhance production efficiency. By swiftly processing and analyzing extensive datasets, companies can expeditiously access information on market shifts and consumer preferences, promptly adapting their strategies and product development trajectory, thereby elevating productivity and competitiveness. Secondly, it reveals business value. Dynamic analysis can delve deeply into voluminous datasets, unveiling hidden business value concealed within the data. Businesses can adeptly seize opportunities by analyzing market trends, consumer behavior data, executing precise marketing and promotion strategies, and attaining heightened profits and benefits. Thirdly, it fosters personalized services. Dynamic analytical technology, leveraging meticulous data analysis, can furnish users with personalized products and services. Businesses can enhance user satisfaction and loyalty by tailoring product recommendations and personalized marketing services based on user needs, preferences, and historical behavior data. Lastly, it fortifies risk management. Dynamic analysis, via real-time monitoring and analysis of big data, assists businesses in promptly discerning potential risks and implementing corresponding countermeasures. Companies can deploy dynamic analysis technology to formulate risk warning models, enabling real-time monitoring and analysis of market fluctuations, competitors, supply chains, and more, thereby mitigating operational risks.

Technological innovation plays a crucial role in the digital economy’s developmental trajectory. With the application of these advanced technologies, fundamental changes are occurring in the business models of the digital economy. New business models, such as AI-based personalized services and secure transactions facilitated by blockchain technology, are gradually emerging and propelling economic growth. Technological innovation enhances operational efficiency in the digital economy. Through automation and intelligence, enterprises can more effectively manage resources, improve productivity, and reduce operational costs. With the rapid development of technology, the digital economy faces unprecedented market opportunities. However, this also brings about new challenges, such as the need for continuous investment in technology, talent training, and integration with the traditional economy.

In summary, dynamic analysis in the digital economy is multifaceted, encompassing productivity enhancement, business value discovery, personalized service creation, and reinforced risk management. Through dynamic analysis facilitated by technological innovation, businesses can adeptly navigate the digital economy environment, enhancing their competitiveness and operational benefits. Moreover, technological innovation assumes a pivotal role in the digital economy, particularly within the framework of the green economy. In this context, technological innovation propels the dynamic development of the digital economy, fostering a symbiotic relationship between economic benefits and environmental sustainability. This synergy is achieved through transformative shifts in industrial structure, innovative production methods, and the delicate balance between economic growth and environmental protection.

### Experimental design

The dataset chosen for the experiment is sourced from the World Bank Open Data, an online platform providing comprehensive data on various aspects such as economics, population, education, and the environment for countries globally. This dataset is a valuable and extensive resource for research and applications in political and economic analysis and development research. Interested individuals can download the dataset from the following website: https://databank.worldbank.org/home.aspx. The dataset covers data from more than 200 countries and territories worldwide, including high-, middle-, and low-income countries. The collection spans a wide range of periods, covering historical data and statistics from 1960 to the most recent year. This period and geographic coverage make the valuable dataset for analyzing current economic and social conditions and studying long-term trends and patterns. The selection of variables in this study is shown in Table 4:

**Table 4:** Experimental variable.

Variable category	Name	Description
Green economy	Environmental protection expenditure	The total expenditure of a country or region on environmental protection measures reflects the financial investment in environmental protection.
	Renewable energy consumption ratio	The percentage of renewable energy in a country or region’s total energy consumption, which measures the extent of sustainable energy consumption.
	Carbon dioxide emissions	The overall carbon dioxide emissions of a country or region are an important indicator of the contribution of environmental pollution and climate change.
Technological innovation	Research and development (R&D) expenses	Total national or regional spending on R&D reflects financial investment in innovative activities.
	Number of patent applications	It represents the total number of patent applications filed by a country or region in a certain period and is used to measure technological innovation activity.
Digital economy	Internet penetration	It refers to the percentage of a country or region’s population using the Internet and measures the prevalence of digital technology.
	Digital payment usage rate	The proportion of a country or region’s population using digital payment methods reflects the popularity and acceptance of digital financial services.
Traditional production	Output value of traditional manufacturing industry	The total output value of a country or region’s traditional manufacturing industry reflects the economic scale of the traditional manufacturing industry.
	Traditional agricultural output	The total output of traditional agriculture in a country or region measures the economic contribution of the traditional agricultural sector.

In this study, a total of 2000 sample data points were systematically selected and subsequently partitioned into four distinct datasets: Dataset 1, Dataset 2, Dataset 3, and Dataset 4. The hardware configuration for the experiment to proceed smoothly included a processor equipped with Intel CPU E5-2620 v4 @ 2.10GHz and an NVIDIA Titan Xp 12GB GPU with 128 GB of memory. This hardware configuration ensures that the experiment has sufficient computing power and memory resources to handle the complex data analysis and model training process. Regarding software, the experimental environment was based on the Ubuntu 16.04 LTS operating system and used the PyTorch 1.7.0 deep learning (DL) framework. PyTorch was a flexible and powerful DL library that provided the necessary computational and model development tools for experimentation. The stability and openness of the Ubuntu operating system, coupled with the efficiency and ease of use of the PyTorch framework, form the software basis of this experiment. In this experiment, the learning rate was established at 0.01, adopting a batch size of 16 over 500 iterations. The hidden layer comprised 100 nodes, with the chosen activation function being Sigmoid. The comparative models integrated into the experiment encompassed Linear Regression (LR), Decision Tree (DT), Random Forest (RF), Support Vector Machine (SVM), and Naive Bayes (NB) models. Presented herewith is a segment of the code instrumental to the experimental methodology:

Import the required library

Import Pandas as PD # for data processing and analysis

Import numpy as np # for numerical calculations

Import requests # for network requests

Import JSON # Used to process JSON data

## Dynamic impact analysis of technological innovation on the digital economy and sustainable strategy optimization

### Performance comparison analysis of dynamic analysis models

**Fig 2:**
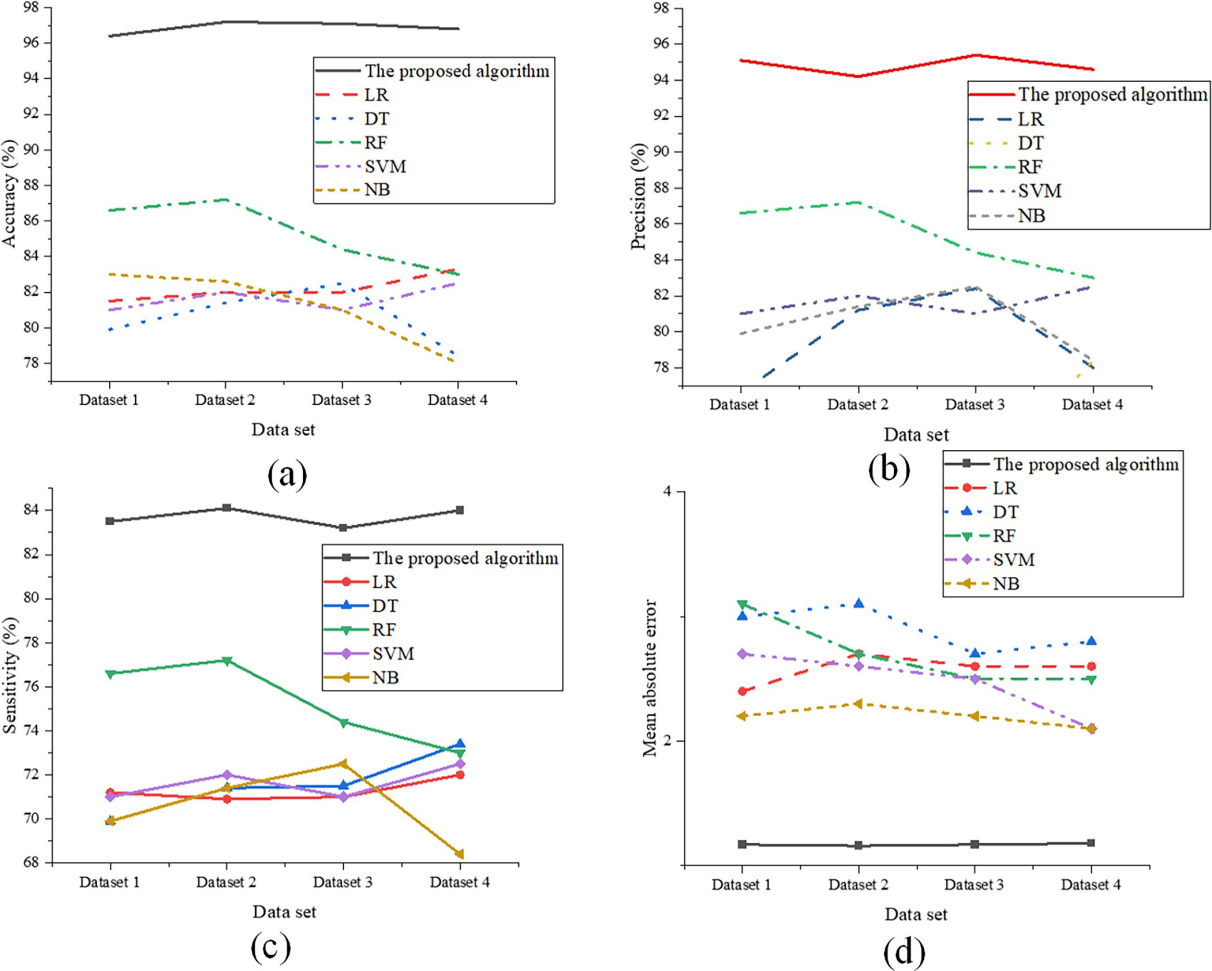
Comparison Results of Model Performance (a) Comparative analysis of Accuracy; (b) Comparative analysis of Precision; (c) Comparative analysis of Sensitivity; (d) Comparative analysis of Mean Absolute Error (MAE).

The data from the four datasets are input into the optimized and comparative models in this study. The experimental results are presented in Fig 2:

In Fig 2, the optimized model proposed in this study displayed notable enhancements in accuracy, precision, sensitivity, and MAE when forecasting future trends compared to conventional models. Primarily, the proposed optimized model achieved a prediction accuracy of 97.2%, demonstrating high precision on a testing dataset comprising 500 samples. Relative to traditional models, the proposed optimized model showcased an average improvement of 14.6%, attesting to the efficacy of the proposed enhancements in augmenting prediction accuracy. Secondly, the proposed optimized model exhibited considerable refinement in precision, signifying the model’s accuracy in classifying sample data. In this regard, the proposed optimized model attained a precision of 95.4%, denoting elevated classification accuracy on the 500-sample testing dataset. In contrast to traditional models, the proposed model averaged a 19.2% enhancement, further affirming the efficacy of the proposed optimization in classification tasks. Moreover, the proposed optimized model demonstrated noteworthy progress in sensitivity, reflecting the model’s proficiency in identifying positive samples. Achieving an 84.1% sensitivity on the 500-sample testing dataset, the proposed optimized model averaged an 11.8% improvement over traditional models. This data underscores the heightened sensitivity of the proposed optimization method in recognizing positive samples. Lastly, an evaluation of the MAE metric, quantifying the average disparity between the model’s predicted outcomes and the actual values, revealed satisfactory results for the proposed optimized model, with an MAE of merely 1.16. In comparison to traditional models, the proposed optimized model averaged a reduction of 1.4, further substantiating the effectiveness of the proposed optimization method in predictive tasks.

### Dynamic impact of digital economy under the background of technological innovation

**Fig 3:**
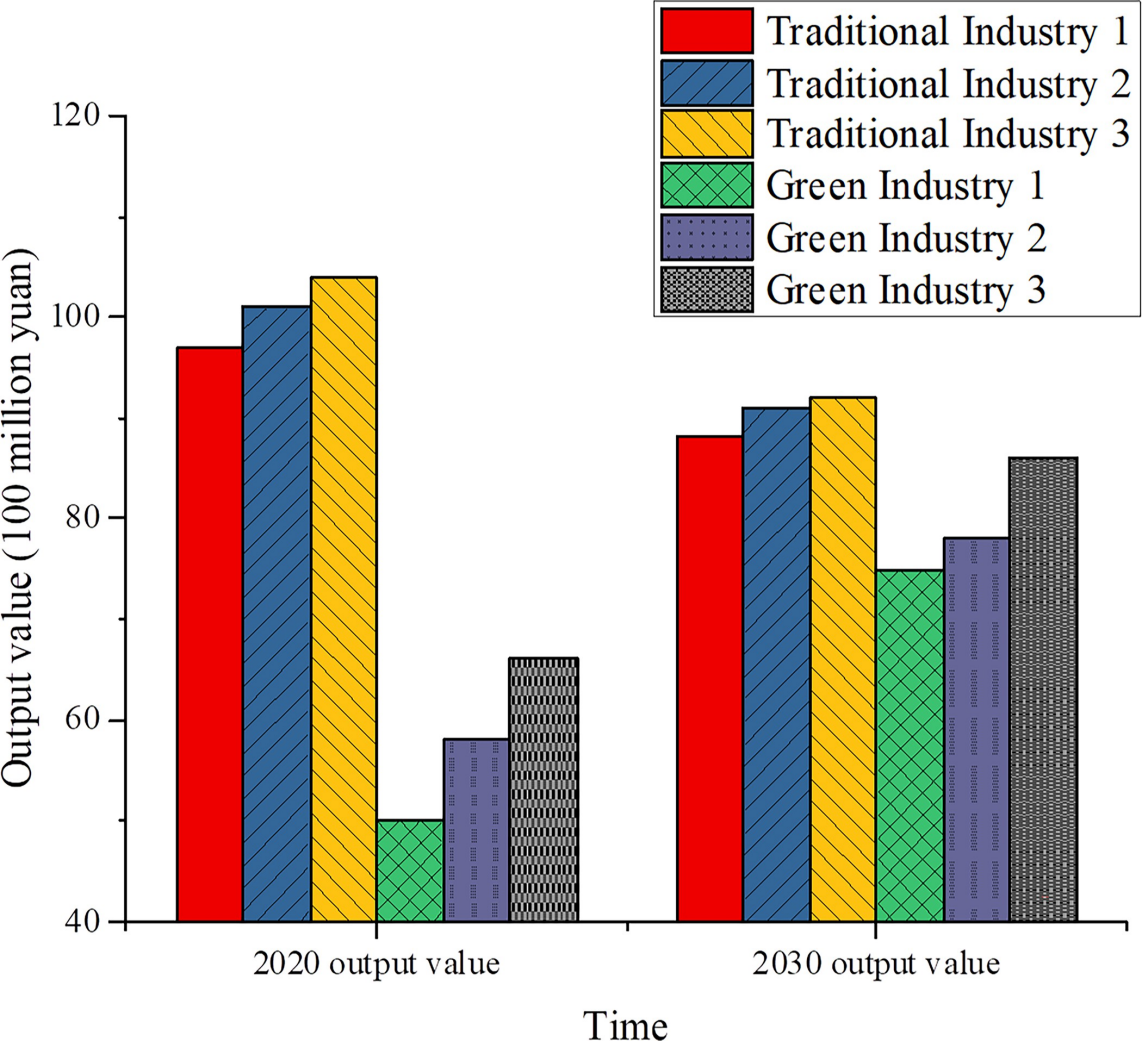
Comparative results of industrial output forecast.

**Fig 4:**
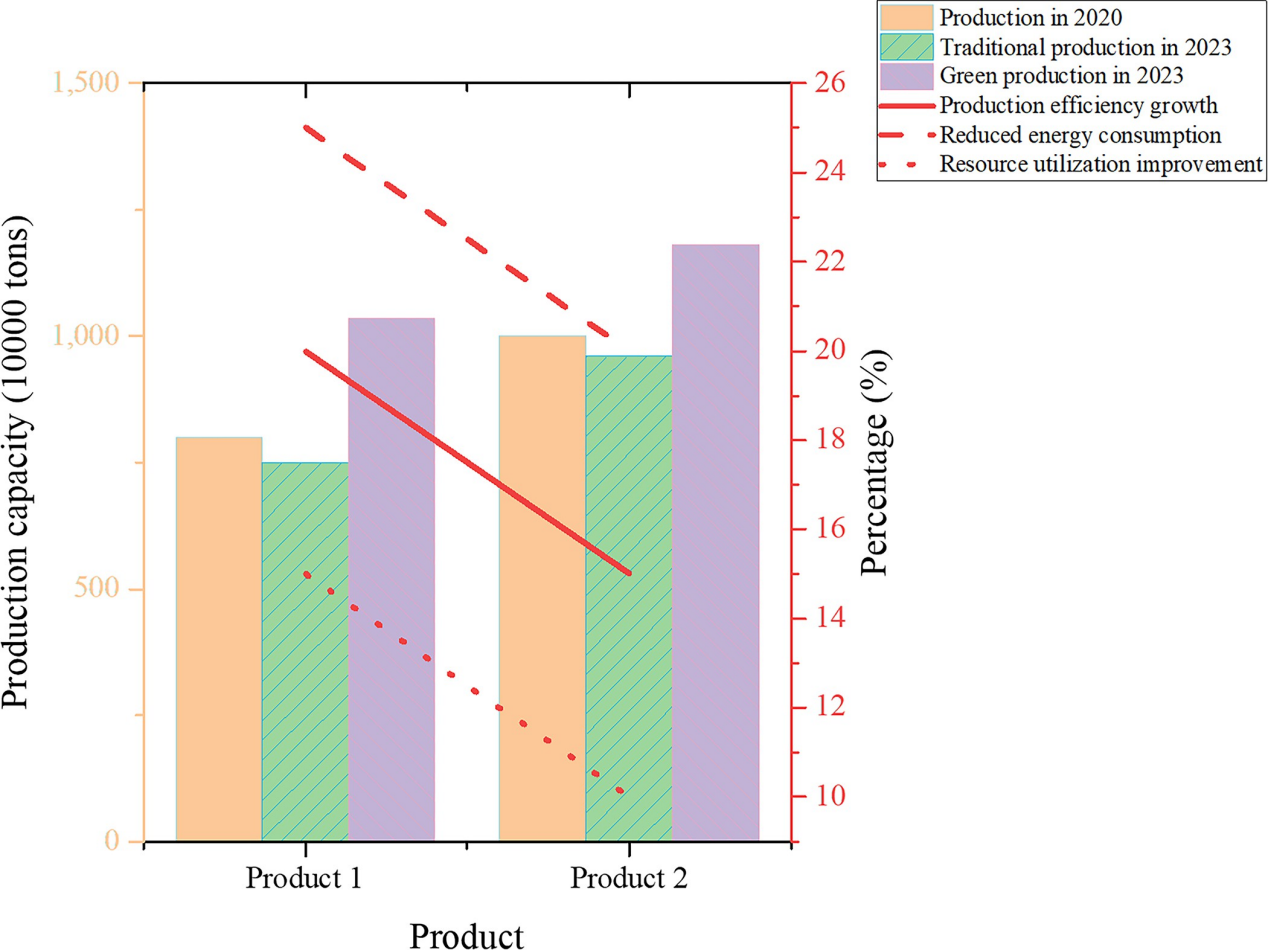
Comparative results of production forecast under different production methods.

Within the framework of the green economy, technological innovation has exerted a notable influence on alterations in industrial structure. The progression of the digital economy has propelled the metamorphosis and enhancement of diverse industries, progressively supplanting conventional high-energy and high-pollution sectors with environmentally sustainable green industries, thereby fostering the prosperity of nascent sectors. The experiment encompassed an examination of 2020 data pertaining to three conventional industries and three green industries. Through the anticipation of pertinent indicators for 2030 and subsequent comparisons, traditional industries mainly include coal mining and processing, chemical manufacturing, and steel manufacturing. These industries are considered typical representatives of high energy consumption and high pollution due to their substantial energy consumption and emissions of pollutants in the production process. Coal mining and processing involve extracting and processing underground or surface coal resources. Chemical manufacturing encompasses the production of various chemical substances and products, a process that may threaten the environment and human health. Steel manufacturing is a crucial foundational industry that transforms iron ore into steel products, serving as a major source of energy consumption and carbon emissions. In contrast, green industries include solar power generation, wind power generation, and the manufacturing of energy-saving and environmental protection equipment. These industries utilize renewable energy and advanced technologies to drive the transformation of the energy structure, reduce GHG emissions, and promote sustainable economic development. Solar power generation and wind power generation, as essential sources of clean energy, are experiencing technological advancements and cost reductions that position them as key industries in addressing climate change. The manufacturing of energy-saving and environmental protection equipment focuses on producing equipment for reducing energy consumption, controlling pollution, and recycling resources, playing a crucial role in enhancing environmental performance across various industries and sectors of life. The experimental findings are elucidated in Fig 3:

In Fig 3, it can be anticipated that the future industrial structure will undergo significant changes. These changes align with the principles and development trajectory of the green economy, further promoting sustainable development. Firstly, regarding the forecast of the output value of traditional industries, based on data analysis from 2020, it is expected that the output value of traditional industries will decline. Specifically, the average output value of traditional industries is projected to decrease by 11.4 billion RMB. This data suggests that traditional industries face challenges in output reduction influenced by market demand, technological progress, and other limiting factors. Secondly, in terms of the expected output value of green industries, green industries are expected to achieve output expansion due to the advancement of the digital economy. In particular, the average output value of green industries is projected to increase by 23.4 billion RMB. Technological innovation within the green economy has driven advancements in production methods, triggering a revolutionary transformation in traditional production methods. The development of digital technologies, including big data analytics, IoT, and AI applications, has facilitated process optimization and efficiency improvement. This analysis not only reveals the positive role of technological innovation in the transformation of industrial structure under the backdrop of the green economy but also demonstrates the importance of green industries in promoting sustainable development. With technological progress, green industries can more effectively meet market demands while reducing negative environmental impacts, marking a shift towards more sustainable production and consumption patterns. These findings emphasize the importance of prioritizing and investing in green technologies and industries when formulating future economic development strategies. The experiment juxtaposed the output of two products under traditional and green production methodologies, with the results delineated in Fig 4.

Fig 4 illustrates that by adopting innovative digital production methods, the green industry has not only achieved a significant increase in output, with an average growth of 2.135 million tons but also experienced a substantial improvement in production efficiency. This observation emphasizes that innovative digital production methods can bring substantial benefits to the economy, positively influencing the sustainable development of the digital economy. Innovative digital production methods have enhanced the production capacity of the green industry, highlighting the crucial role played by digital production innovation. These innovations contribute to increased efficiency in the production process and facilitate the rational use of resources and energy. By introducing advanced digital technologies and intelligent systems, the green industry can adeptly manage and control the production process, thereby reducing production costs and improving the quality of products and services. In addition to the enhancement of economic benefits, the innovation in digital production methods has also positively impacted aspects such as business model innovation, market space expansion, and industrial structure transformation. This innovation provides additional business opportunities for the green industry. Leveraging digital technology, the green industry can explore new business models, such as the sharing economy and platform economy, promoting resource sharing, collaborative innovation, and flexible operations, thereby increasing economic benefits and creativity. Simultaneously, improving the quality and efficiency of green products and services, aligning with consumer demands for environmental protection and sustainable development, expands market space. The innovation in digital production methods enables the green industry to enter the global market, increase exports, foster collaboration with other industries, and enhance market competitiveness and influence. Furthermore, this innovation accelerates the upgrade and transformation of the green industry, promoting rapid adjustments and optimizations in industrial structure. Through the application of digital technology, the green industry can achieve intelligent manufacturing, automated production, and more, enhancing production efficiency and resource utilization efficiency, thus propelling the green industry towards a high-value, innovation-driven trajectory.

### Sustainable development strategies based on dynamic analysis

A series of strategies need to be formulated to drive reform and transformation in various areas to achieve sustainable development. These strategies are detailed in Table 5:

**Table 5:** Sustainable development strategies.

Strategy	Specific analysis
Strengthen the integration of green technology and digital technology	It combines green technology innovation and digital development to promote the application of green smart manufacturing and clean energy technology. Strengthen the role of digital technology in energy efficiency improvement and environmental monitoring.
Optimize the digital economic policy environment.	It formulates policies that encourage the development of the digital economy, such as digital tax incentives, data security, and privacy protection regulations while ensuring that these policies are coordinated with green economic goals.
Improving the application of the digital economy in environmental management	It uses digital technologies such as big data and the IoT to optimize environmental management, such as improving resource efficiency and reducing waste and pollution emissions through intelligent monitoring systems.
Promote international cooperation and exchanges in the fields of green and digital technologies	It strengthens cross-border cooperation and shares green and digital technology innovations, especially in sustainable energy, smart cities, and environmental protection.

These strategies form an essential element of sustainable development grounded in dynamic analysis. Nevertheless, their application requires careful adaptation and implementation in accordance with the unique circumstances of individual countries and regions. Effective collaboration among governments, businesses, and the public is imperative, necessitating collective efforts to attain sustainable development goals and progress toward a more sustainable future.

## Discussion

Experiments are undertaken to optimize model construction utilizing a dataset comprising 500 samples. The model’s predictive performance for future trends is assessed using accuracy, precision, sensitivity, and MAE metrics. The optimized model demonstrates remarkable outcomes across these metrics, achieving a high level of performance. Specifically, the model exhibits accuracy, precision, sensitivity, and MAE of 97.2%, 95.4%, 84.1%, and 1.16, respectively. These results affirm the effectiveness of the optimized model in prediction, showcasing a significant improvement compared to conventional models. The optimization methodology outlined in this study presents a more precise and reliable solution to challenges in forecasting future trends. In examining changes in industrial structure, the findings suggest a projected decrease of 11.4 billion yuan in the average output value of traditional industries. Conversely, the average output value of green industries is anticipated to rise by 23.4 billion yuan. Green industries align with the trends and requirements of the digital economy and benefit from factors such as environmental preservation and sustainable development. This growth in green industries is consistent with the principles of the green economy and holds positive implications for achieving sustainability. The discourse highlights that innovation in digital production methods positively influences the sustainable development of the digital economy. This innovation introduces more efficient, environmentally friendly, and sustainable production methods and technological solutions for green industries, providing support and momentum for their sustainable development. Comparative results derived from 2020 data confirm the constructive role of digital production method innovation at the economic level and contribute to the attainment of sustainable development goals in the digital economy. In previous studies, the study by Johnson and Smith (2021) primarily employed qualitative analysis methods, focusing on constructing theoretical frameworks (Johnson and Smith, 2021, [34]). In contrast, this study utilizes a data-driven MLP network model, enabling quantitative analysis and in-depth data mining, thereby providing more specific and quantified research results. While the study by Williams and Davis (2021) touched upon the digital economy, it mainly concentrated on short-term impact analysis (Williams and Davis, 2021, [35]). In comparison, this study analyzes short-term impacts and extends its scope to long-term impacts and sustainable development strategies, offering a more comprehensive perspective.

The outcomes suggest that the optimized model, examination of industrial structural changes, and investigation into production method innovation collectively illustrate the positive influence of digital production method innovation in the digital economy. These findings offer a more accurate and dependable approach to realizing economic sustainability, fostering the expansion of green industries, and advancing the digital economy—all grounded in environmental protection and sustainability principles.

## Conclusion

The green economy is acknowledged as a pivotal avenue toward achieving sustainable development. Additionally, the evolution of the digital economy has brought about substantial transformations in conventional economic paradigms. This study endeavors to scrutinize the dynamic influence of technological innovation on the digital economy. Initially, the constraints of conventional DL networks are examined, and optimization methods for model enhancement are proposed. Subsequently, optimization strategies for MLP networks are investigated, focusing on assessing the digital economy’s impact through the equilibrium of changes in industrial structure, innovations in production modes, and the ramifications on economic growth and the environment through feature analysis. Ultimately, the model’s predictive reliability is substantiated through performance benchmarks against alternative models, accompanied by the formulation of pertinent sustainable development strategies. Experimental findings revealed that the optimized model attained an accuracy of 97.2%, precision of 95.4%, and sensitivity of 84.1%, reflecting average improvements of 14.6%, 19.2%, and 11.8%, respectively, compared to traditional models. The MAE stood at a mere 1.16, indicating an average reduction of 1.4 when juxtaposed with traditional models. In exploring shifts in industrial structure, the average output of traditional industries witnessed a decline of 11.4 billion yuan. Conversely, propelled by the digital economy, the anticipated average output of green industries exhibited an increase of 23.4 billion yuan. In investigating production mode innovation, green industries registered heightened production levels and markedly enhanced production efficiency, showcasing an average growth of 2.135 million tons. This study extends the theoretical framework of the integration between the digital economy and the green economy, proposing a novel perspective to comprehend the role of technological innovation in fostering sustainable economic development. By introducing an optimized MLP network model, this study innovates methodologically and enhances model accuracy and efficiency by comprehensively considering energy efficiency and data processing technologies. Simultaneously incorporating theories and methods from various disciplines, such as economics, environmental science, and computer science, the study illustrates the significance and efficacy of interdisciplinary research in addressing complex economic and environmental issues. The study findings offer policymakers a scientific basis, aiding them in better understanding how technological innovation influences the digital economy under the backdrop of the green economy and formulating effective economic and environmental policies accordingly. While this study utilized the World Bank Open Data dataset, providing big data resources, the scope and sample size of the dataset may still pose limitations. Specifically, the dataset might not fully cover or accurately reflect certain countries or regions with unique circumstances. Additionally, although the optimized MLP network model employed in this study performed well in handling the provided data, its general applicability and effectiveness in diverse contexts may require further validation. The study predominantly relies on quantitative analysis, potentially falling short of fully revealing the impact of certain qualitative factors, such as policy changes and market psychology, on the green economy and digital economy.

In future research, there should be a more in-depth exploration of the pivotal role of digital technology in facilitating industrial structural upgrades. With rapid technological advancements, particularly breakthroughs in big data, AI, and cloud computing, digital technology has emerged as a potent force propelling industrial transformation. Future studies should analyze how digital technology aids traditional industries in achieving digital transformation, enhancing production efficiency and environmental friendliness, particularly in crucial sectors like energy, manufacturing, and services. Furthermore, research should delve into how digital technology fosters the development of emerging industries, especially those closely tied to the green economy and sustainable development. Recent challenges have underscored the importance of digital green innovation in the integrated green building supply chain. Therefore, future research should examine how digital technology plays a role in various stages of green building projects, including design, construction, operation, and maintenance. In particular, research should emphasize the potential of digital technology in promoting supply chain efficiency, cost reduction, and sustainability. For instance, IoT and intelligent analytics tools can better monitor and manage energy usage, reduce waste generation, and enhance resource utilization efficiency. Additionally, research should explore the role of digital technology in promoting collaboration and communication among various parties in the supply chain and how these technologies can address rapid environmental changes and market demand fluctuations.

### Managerial implication

The findings of this study hold significant managerial implications for policymakers, business executives, and relevant stakeholders.

For policymakers, emphasis should be placed on promoting the application of digital technology across various industries, mainly by providing support for the digital transformation of traditional sectors. This support could be facilitated through measures such as financial and tax incentives, as well as technological training to encourage businesses to adopt advanced digital technologies. Additionally, increased investment in the green economy is recommended. Policymakers should formulate and implement policies to foster environmental protection and sustainable development. This may involve increasing support for studying and developing green products and technologies and incentivizing green consumption. For business executives, recognizing the importance of digital technology in enhancing production efficiency, reducing costs, and strengthening market competitiveness is crucial. Leveraging digital technology for data analysis and decision support can help companies accurately understand market demands and optimize operational management. Simultaneously, incorporating green development as a strategic direction is advised for its positive environmental impact and potential value in current and future markets. Companies can enhance their brand value and market competitiveness by implementing green production processes and products. For stakeholders, collaboration is essential in the context of the digital and green economies. All parties in the industrial chain should strengthen cooperation to explore and implement solutions for digitization and green initiatives. Establishing partnerships allows for sharing resources, creating greater social and economic value. Investors should focus on businesses and projects dedicated to digitization and the green economy. Such investments can yield economic returns while driving sustainable societal development.

By implementing the above recommendations, a harmonious unity of economic growth, environmental protection, and social well-being can be achieved within the broader context of digital and green economies.

### Practical implications

The outcomes of this study contribute not only to theoretical advancements but also hold profound significance at the practical and societal levels.

The study underscores the crucial role of technological innovation and digitization in promoting social sustainability. By employing green technology and digital means, it becomes possible to effectively reduce environmental pollution, enhance resource utilization efficiency, and foster the coordinated development of economic growth and environmental protection. The study findings can serve as a scientific basis for government departments to formulate relevant environmental protection and sustainable development policies, guiding the general public to pay greater attention to environmentally friendly lifestyles. In the era of the digital economy, this study provides direction and strategies for businesses to undergo transformation and upgrade, particularly in adopting emerging digital technologies and implementing green production. This will assist companies in improving efficiency, reducing costs, and enhancing competitiveness in the market. For emerging businesses and entrepreneurs, the study findings can serve as a reference for market exploration and product strategy design, especially in green technology and sustainable business models.

The exploration of green economy and technological innovation in this study positively impacts public awareness and engagement in environmental protection. The public can contribute to environmental protection and sustainable development through more environmentally friendly consumption choices and lifestyles. Various sectors of society, including educational institutions and non-governmental organizations, can utilize these research findings for public education and advocacy activities, promoting green living. The insights and recommendations presented in this study can encourage policymakers to consider more comprehensive factors when formulating economic and environmental policies, particularly those related to fostering technological innovation and digital transformation. Policy changes and implementation will create a healthier and more sustainable economic environment, setting the stage for better living conditions for future generations.

Through the practical and societal applications outlined above, the outcomes of this study can drive economic and technological development and promote overall social sustainability and environmental protection, laying the foundation for creating a more harmonious future society.

## Supporting information

S1 Data (ZIP)
