# Supplementary material for: The impact of technological innovation on the green digital economy and development strategies
Source: PLoS One. 2024 Apr 25;19(4):e0301051. doi: 10.1371/journal.pone.0301051 (PMC11045117; doi:10.1371/journal.pone.0301051)
Supplement: S1 Data — (ZIP) [file pone.0301051.s001.zip › data packet/Code Description.docx]

1. Import Required Libraries

-Import the pandas library for data processing and analysis.

-Import the numpy library for numerical computation.

-Import the requests library for sending network requests.

-Import the JSON library for handling JSON data.

-Import the StandardScaler class for data standardization.

-Import the SelectKBest class for feature selection.

-Data Collection Function collect_data()

2. Data collection function collect_data()

-collect_data() is a defined function used for data collection.

-In this function, use the requests library to send a GET request to the address 'http://api.example.com/data' and store the response in the response variable.

-Then, use the loads() function from the JSON library to convert the response.text into JSON-format data and store the result in the data variable.

-Finally, return the collected data data as the function's output.

3. Data Collection

-Call the collect_data() function to retrieve data and store the returned data in the data variable.

4. Data Preprocessing Function preprocess_data(data)

-preprocess_data() is a defined function used for data preprocessing.

-In this function, first, convert the incoming data into DataFrame format and store the transformed result in the df variable.

-Then, use the dropna() function to remove rows containing missing values, resulting in a DataFrame with missing values removed.

-Next, you can perform steps of feature engineering, such as adding new features, handling date data, etc. (not specifically demonstrated here).

-Then, create a StandardScaler object scaler for data standardization.

-Use the fit_transform() method of scaler to standardize the DataFrame with missing values removed and store the result in the scaled_data variable.

-Create a SelectKBest object selector with a specified k value of 10 for feature selection.

-Use the fit_transform() method of the selector to perform feature selection on the standardized data scaled_data, simultaneously passing the 'target' column from df as the target variable.

-Finally, return the data after feature selection as selected_data.

5. Data Preprocessing

-Call the preprocess_data() function, pass the previously collected data as a parameter, and store the preprocessed data in the preprocessed_data variable.

6. Import Required Libraries

-Import the LinearRegression class for constructing linear regression models.

-Import the RandomForestRegressor class for constructing random forest regression models.

-Import the GridSearchCV class for model parameter tuning.

7. Model Building Function build_model()

-build_model() is a defined function used for model construction.

-In this function, instantiate the LinearRegression class and store the instance in the linear_model variable, creating a linear regression model object.

-Then, instantiate the RandomForestRegressor class and store the instance in the rf_model variable, creating a random forest regression model object.

-Add other logic for constructing models as needed (not specifically demonstrated here).

-Finally, place the instantiated model objects into a list and return them as the function's output.

8. Model Optimization Function optimize_model(models, data)

-optimize_model() is a defined function used for model optimization.

-In this function, first, create an empty list of optimized_models for storing optimized models.

-Then, use a for loop to iterate through each model in the input models list.

-Within the loop, first set the parameter candidate values param_grid, including three parameters: 'n_estimators', 'max_depth', and 'min_samples_split', each with different ranges.

-Next, use the GridSearchCV class for model parameter tuning. Pass the current model object model and the parameter candidate values param_grid into the GridSearchCV class, and set the cv parameter to 5 (indicating 5-fold cross-validation).

-Call the fit() method of grid_search, passing the feature data X and target data y from the input data for model parameter tuning.

-Get the best model, i.e., call the best_estimator_ attribute of grid_search, and store the best model in the optimized_model variable.

-Add the optimized_model to the optimized_models list.

-After completing the loop, return the optimized_models list containing the optimized models as the function's output.

9. Model Construction

-Call the previously defined build_model() function to generate a list of models and store them in the models variable.

10. Import Required Libraries

-Import functions such as accuracy_score, precision_score, recall_score, and mean_absolute_error from the sklearn.metrics library for model performance evaluation.

11. Predict Future Trend Function predict_future_trend(model)

-predict_future_trend() is a defined function used for predicting future trends.

-In this function, use the passed model to call the predict() method, providing future data future_data for prediction.

-Store the prediction result in the future_trend variable and return it as the function's output.

12. Model Performance Evaluation Function calculate_accuracy(y_true, y_pred)

-calculate_accuracy() is a defined function used to calculate the accuracy of a model.

-In this function, use the accuracy_score function, passing the true labels y_true and predicted labels y_pred to calculate accuracy.

-Store the accuracy result in the accuracy variable and return it as the function's output.

13. Model Performance Evaluation Function calculate_precision(y_true, y_pred)

-calculate_precision() is a defined function used to calculate the precision of a model.

-In this function, use the precision_score function, passing the true labels y_true and predicted labels y_pred to calculate the precision.

-Store the precision result in the precision variable and return it as the function's output.

14. Model Performance Evaluation Function calculate_sensitivity(y_true, y_pred)

-calculate_sensitivity() is a defined function used to calculate a model's sensitivity (recall).

-In this function, use the recall_score function, passing the true labels y_true and predicted labels y_pred to calculate sensitivity.

-Store the sensitivity result in the sensitivity variable and return it as the function's output.

15. Model Performance Evaluation Function calculate_absolute_error(y_true, y_pred)

-calculate_absolute_error() is a defined function used to calculate the absolute error of a model.

-In this function, use the mean_absolute_error function, passing the true values y_true and predicted values y_pred to calculate the absolute error.

-Store the absolute error result in the absolute_error variable and return it as the function's output.

16. Predict Future Trends

-Call the previously defined predict_future_trend() function, passing the optimized model optimized_model, to predict future trends.

-Store the prediction result in the future_trend variable.

17. Model Performance Evaluation

-Call the previously defined calculate_accuracy() function, passing the target values from the preprocessed data preprocessed_data and the optimized model optimized_model to evaluate the prediction results and calculate accuracy.

-Store the accuracy result in the accuracy variable.

-Similarly, call the calculate_precision(), calculate_sensitivity(), and calculate_absolute_error() functions to calculate precision, sensitivity, and absolute error, storing the results in the precision, sensitivity, and absolute_error variables, respectively.

18. Import Required Libraries

-Import the pandas library for data processing and analysis.

-Import the matplotlib.pyplot library for data visualization.

19. Analyze Industry Structure Change Function analyze_industry_change(data)

-analyze_industry_change() is a defined function used to analyze changes in industry structure.

-In this function, first group the data by year, using the groupby () method, grouping the data based on the 'year' column, and then calculating the average industry structure for each year.

-Store the calculation results in the industry_avg variable.

20. Plot Line Graph to Show Industry Structure Change

-Create a figure with a size of 10x6 using the statement plt.figure(figsize=(10, 6)).

-Use the plt. plot() function to plot a line graph, passing the index of industry_avg as the x-axis data and the values of industry_avg as the y-axis data.

-Use plt. xlabel() to set the x-axis label as 'Year'.

-Use plt. ylabel() to set the y-axis label as 'Industry Structure'.

-Use plt. title() to set the graph title as 'Changes in Industry Structure Over Time'.

-Use plt.show() to display the graph.

21. Import Required Libraries

-Import the pandas library for data processing and analysis.

-Import the matplotlib.pyplot library for data visualization.

22. Analyze Industry Structure Change

-Call the previously defined analyze_industry_change() function, passing the preprocessed data preprocessed_data for analyzing industry structure.

-Store the results in the industry_change variable (this variable is ignored in this context).

23. Generate Precise JSON Code Using ECharts Syntax

-Add the other parts of JSON code from the provided ECharts template.

-At the end, add "toolbox": {"feature": {"saveAsImage": {}}} to include a toolbar for saving images in the graph.

24. Analyze Production Innovation Function analyze_production_innovation(data)

-analyze_production_innovation() is a defined function used to analyze production innovation.

-In this function, first group, the data by year, using the groupby() method, grouping the data based on the 'year' column, and then calculate the average value of the production innovation index for each year.

-Store the calculation results in the innovation_avg variable.

25. Plot Bar Chart to Show Production Innovation

-Create a figure with a size of 10x6 using the statement plt.figure(figsize=(10, 6).

-Use the plt.bar() function to plot a bar chart, passing the index of innovation_avg as the x-axis data and the values of innovation_avg as the y-axis data.

-Use plt.xlabel() to set the x-axis label as 'Year'.

-Use plt.ylabel() to set the y-axis label as 'Production Innovation'.

-Use plt.title() to set the graph title as 'Production Innovation Over Time'.

-Use plt.show() to display the graph.

26. Import Required Libraries

-Import the pandas library for data processing and analysis.

27. Analyze Production Innovation

-Call the previously defined analyze_production_innovation() function, passing the preprocessed data preprocessed_data for analyzing production innovation.

-Store the results in the production_innovation variable (this variable is ignored in this context).

28. Generate Precise JSON Code Using ECharts Syntax

-Add the other parts of JSON code from the provided ECharts template.

-At the end, add "toolbox": { "feature": { "saveAsImage": {} } } to include a toolbar for saving images in the graph.

29. Develop Sustainable Development Strategy Function develop_sustainable_strategy(data)

-develop_sustainable_strategy() is a defined function used for analyzing and formulating sustainable development strategies based on preprocessed data.

-In this function, an example of strategy analysis and formulation is provided.

-Example: Calculate the Industrial Environmental Impact Index by dividing the 'emissions' column by the 'production' column to obtain the 'environmental_impact' column, representing the industrial environmental impact index.

-Example: Formulate strategies based on the environmental impact index by assessing the average value of the 'environmental_impact' column. If it is greater than 0.5, the strategy is formulated as 'Enhance environmental protection measures and reduce emissions'; otherwise, the strategy is 'Encourage innovation and develop clean production technologies.'

-Store the formulated strategy in the strategy variable.

-Return the strategy.

30. Call the Function and Pass Data

-Call the develop_sustainable_strategy() function, passing the preprocessed data for the formulation of sustainable development strategies.

-Store the formulated strategy result in the production_innovation variable (this variable is ignored in this context).

31. Import Required Libraries

-Necessary libraries have been imported, but their specific purposes are not explained.

32. Formulate Sustainable Development Strategy

-Call the previously defined develop_sustainable_strategy() function, passing the preprocessed data preprocessed_data.

-Store the strategy result in the sustainable_strategy variable (this variable is ignored in this context).

33. Import Required Libraries

-No specific information is provided regarding the importation of relevant libraries.

34. Implement Sustainable Development Strategy Function implement_strategy(strategy)

-implement_strategy() is a defined function used for implementing a sustainable development strategy.

-In this function, first print information about the strategy being implemented.

-Example: Simulate the process of implementing the strategy using the time.sleep() function to mimic the time required for strategy implementation, assuming it to be 3 seconds.

-Finally, print a message indicating the successful implementation of the strategy.

35. Implement a Sustainable Development Strategy

-Call the implement_strategy() function, passing the sustainable development strategy sustainable_strategy as a parameter.

-Execute the process of implementing the sustainable development strategy and print the corresponding information.

Summary:

This code primarily obtains data through network requests and parses it using the JSON library, ultimately storing the parsed data in the data variable. Additionally, it imports libraries such as pandas, numpy, requests, and JSON to facilitate subsequent data processing and analysis operations. The data preprocessing steps are carried out through the preprocess_data() function. Initially, the raw data is transformed into a DataFrame format, and missing values are removed. Then, feature engineering and standardization are applied, followed by feature selection to obtain the preprocessed data preprocessed_data. The code also imports classes like LinearRegression, RandomForestRegressor, and GridSearchCV from the sklearn library for model construction and parameter optimization. Through the build_model() function, linear regression model objects and random forest regression model objects can be created and stored in a list as a return value. The optimize_model() function is defined for model parameter optimization. It iterates through the provided model list, performs grid search optimization on each model, and selects the best model based on predefined parameter candidate values using GridSearchCV. Finally, the optimized models are stored in a list and returned by the function. Functions for model performance evaluation and prediction are also defined. These functions include predict_future_trend() for predicting future trends, calculate_accuracy() for calculating accuracy, calculate_precision() for calculating precision, calculate_sensitivity() for calculating sensitivity (recall), and calculate_absolute_error() for calculating absolute error. Additionally, these functions depend on previously constructed models and related data. The code also imports several functions from the sklearn.metrics library for model performance evaluation. Future trends are predicted, and model performance is assessed by calling the relevant functions. The optimized model is used to predict future trends, and the results are stored in the future_trend variable. Then, using the preprocessed data preprocessed_data and the optimized model optimized_model, the calculate_accuracy(), calculate_precision(), calculate_sensitivity(), and calculate_absolute_error() functions are called to compute model accuracy, precision, sensitivity, and absolute error. The results are stored in the accuracy, precision, sensitivity, and absolute_error variables. Finally, pandas and matplotlib. pyplot libraries are imported for data processing and visualization. The analyze_industry_change() function is called to analyze changes in industry structure, and a line graph is used to visualize changes in industry structure. Within the function, pandas is used for data processing and calculations, and matplotlib.pyplot is used for data visualization. Finally, precise JSON code is generated using ECharts syntax to save the graph or perform other operations. The analyze_production_innovation() function is called to analyze production innovation, and a bar chart is used to visualize changes in production innovation. Within the function, pandas are used for data processing and calculations, and matplotlib.pyplot is used for data visualization. Again, precise JSON code is generated using ECharts syntax to save the graph or perform other operations. The develop_sustainable_strategy() function is called to analyze and formulate sustainable development strategies based on preprocessed data. Within the function, the example code calculates the industrial environmental impact index and formulates strategies based on this index. The formulated strategy is then returned as a result. When calling this function, preprocessed data needs to be passed as a parameter. The sustainable development strategy is formulated using the develop_sustainable_strategy() function, and the implement_strategy() function is called to implement the sustainable development strategy. Within the implement_strategy() function, the strategy implementation process is simulated by printing information and adding wait time to simulate the delay in the implementation process. When calling this function, the formulated strategy needs to be passed as a parameter.
